# Prenatal Exposure to Nitrate in Drinking Water and Adverse Health Outcomes in the Offspring: a Review of Current Epidemiological Research

**DOI:** 10.1007/s40572-023-00404-9

**Published:** 2023-07-16

**Authors:** Pernille Jul Clemmensen, Jörg Schullehner, Nis Brix, Torben Sigsgaard, Leslie Thomas Stayner, Henrik Albert Kolstad, Cecilia Høst Ramlau-Hansen

**Affiliations:** 1 Department of Public Health, Aarhus University, Aarhus, Denmark; 2 Danish Big Data Centre for Environment and Health (BERTHA), Aarhus University, Aarhus, Denmark; 3 Department of Clinical Genetics, Aarhus University Hospital, Aarhus, Denmark; 4 Cirrau - Centre for Integrated Register-based Research at Aarhus University, Aarhus, Denmark; 5 Division of Epidemiology and Biostatistics, University of Illinois at Chicago, School of Public Health, Chicago, IL USA; 6 Department of Occupational Medicine, Aarhus University Hospital, Aarhus, Denmark; 7 Department of Clinical Medicine, Aarhus University, Aarhus, Denmark

**Keywords:** Nitrate, Drinking water, Prenatal, Pregnancy

## Abstract

**Purpose of Review:**

Recently, several epidemiological studies have investigated whether prenatal exposure to nitrate from drinking water may be harmful to the fetus, even at nitrate levels below the current World Health Organization drinking water standard. The purpose of this review was to give an overview of the newest knowledge on potential health effects of prenatal exposure to nitrate.

**Recent Findings:**

We included 13 epidemiological studies conducted since 2017. Nine studies investigated outcomes appearing around birth, and four studies investigated health outcomes appearing in childhood and young adulthood.

**Summary:**

The reviewed studies showed some indications of higher risk of preterm delivery, lower birth weight, birth defects, and childhood cancer related to prenatal exposure to nitrate. However, the numbers of studies for each outcome were sparse, and some of the results were conflicting. We suggest that there is a need for additional studies and particularly for studies that include information on water consumption patterns, intake of nitrate from diet, and intake of nitrosatable drugs.

## Introduction

Nitrate has been classified by the International Agency for Research on Cancer (IARC) according to its carcinogenicity as probably carcinogenic under conditions that lead to nitrosation [1]. Until recently, the majority of studies on potential effects of nitrate in drinking water have focused on a potential cancer risk in adults [2–4]. In the first months, organogenesis may be disturbed by prenatal exposures, and throughout the fetal period, there is a risk of inducing genetic and epigenetic changes or affecting fetal growth [4, 5]. In addition to outcomes appearing around birth, Barker suggested that adult diseases could originate in fetal life [6], indicating the importance of evaluating exposures in fetal life in relation to adult health.

In areas with nitrate-contaminated drinking water, drinking water may account for the majority of maternal nitrate exposure [2]. Nitrogen that leaches into the water systems from nitrogen fertilizers is an important contributor to nitrate contamination, and the nitrate level in maternal drinking water differs by geography, farming intensity, drinking water source (groundwater or surface water), and the use of private/public water supply systems [3, 7]. Generally, the nitrate levels in public water systems in Europe and the USA are below the maximum contaminant level of nitrate [3••]. The current maximum contaminant level of nitrate in drinking water of 10 mg N/L nitrate-nitrogen in the USA (a standard set by the Environmental Protection Agency corresponding to 44 mg/L nitrate) [8] and 50 mg/L nitrate in EU countries (a standard settled in an EU-directive from 1980) [9] was historically established to protect against the short-term exposure effect of methemoglobinemia in infants (i.e., blue baby syndrome) [2]. In the mid-twentieth century, several case reports indicated a higher risk of blue-baby syndrome in bottle-fed babies (with formula prepared with tap water) who were exposed to high levels of nitrate from tap water. Elevated levels of methemoglobin concentrations were found in infants that consumed water with nitrate concentrations > 45 mg/L with clinical signs of methemoglobinaemia occurring at higher exposure levels [1, 2]. The formation of methemoglobin occurs when nitrite or nitrate that is endogenously reduced to nitrite reacts with hemoglobin and impairs the oxygen carrying capacity. This is particularly a problem in infants where the level of methemoglobin reductase is insufficient and the bacterial endogenous conversion of nitrate to nitrite is higher due to a higher pH in the infant stomach [2]. Nitrite also has the ability to react with amines and amides from the diet and drugs and form *N*-nitroso-compounds (NOCs) [2, 3, 10–13]. Nitrite and nitrate also have been suggested to cross the placental barrier [14, 15]. Epidemiological studies have further linked nitrate exposure to impaired thyroid function probably because nitrate inhibits iodine uptake in the thyroid gland [3••], and animal and experimental studies have suggested that nitrate reduces the production of steroid hormones [2, 16, 17]. Hence, prenatal exposure to nitrate might influence the fetus by several mechanisms, something not taken into account by the current regulatory standards for nitrate.

The potential impact of prenatal exposure to nitrate from drinking water was reviewed in 2006 and 2018 [3, 12]. In 2006, Manassaram et al. concluded that “*Epidemiologic evidence for increased risk for adverse reproductive and developmental outcomes in humans from exposure to nitrate in drinking water is sparse and suggestive at best.*” [12], and the review from 2018 by Ward et al. included nine new studies and concluded that “*Several studies of adverse reproductive outcomes since 2004 have indicated a positive association between maternal prenatal exposure to nitrate concentrations below the MCL (maximum contaminant level) and low birth weight and small for gestational age births. However, most studies did not account for co-exposure to other water contaminants, nor did they adjust for potential risk factors.*” [3••].

The knowledge on health effects of prenatal exposure to nitrate has expanded with numerous epidemiological studies addressing this research question since then, and thus, a new review on the topic is desirable. In this review, we aimed to identify and summarize recent studies published between 2017 and 2022 investigating the association between exposure to nitrate from drinking water in the vulnerable period of fetal life and health effects appearing around birth in addition to health effects appearing later in life.

## Methods

### Search Strategy

We performed a search on three databases (PubMed, Embase, and Scopus) to identify epidemiological studies on the association between prenatal exposure to nitrate from drinking water and health outcomes in the offspring. For the literature search, we used the MESH terms “Pregnant Women,” “Prenatal Exposure Delayed Effects,” “Maternal Exposure,” “Pregnancy”. “Nitrates,” “Drinking Water,” and “Fetus” and EMTREE terms “nitrate,” “pregnancy,” “prenatal exposure,” “maternal exposure,” “pregnant woman,” “fetus,” “perinatal period,” and “drinking water” in addition to the search terms “maternal,” “foetus,” “fetus,” “fetal,” “pregnan*,” “prenatal,” “nitrate*,” “water,” and “drink*.”

**Fig. 1:**
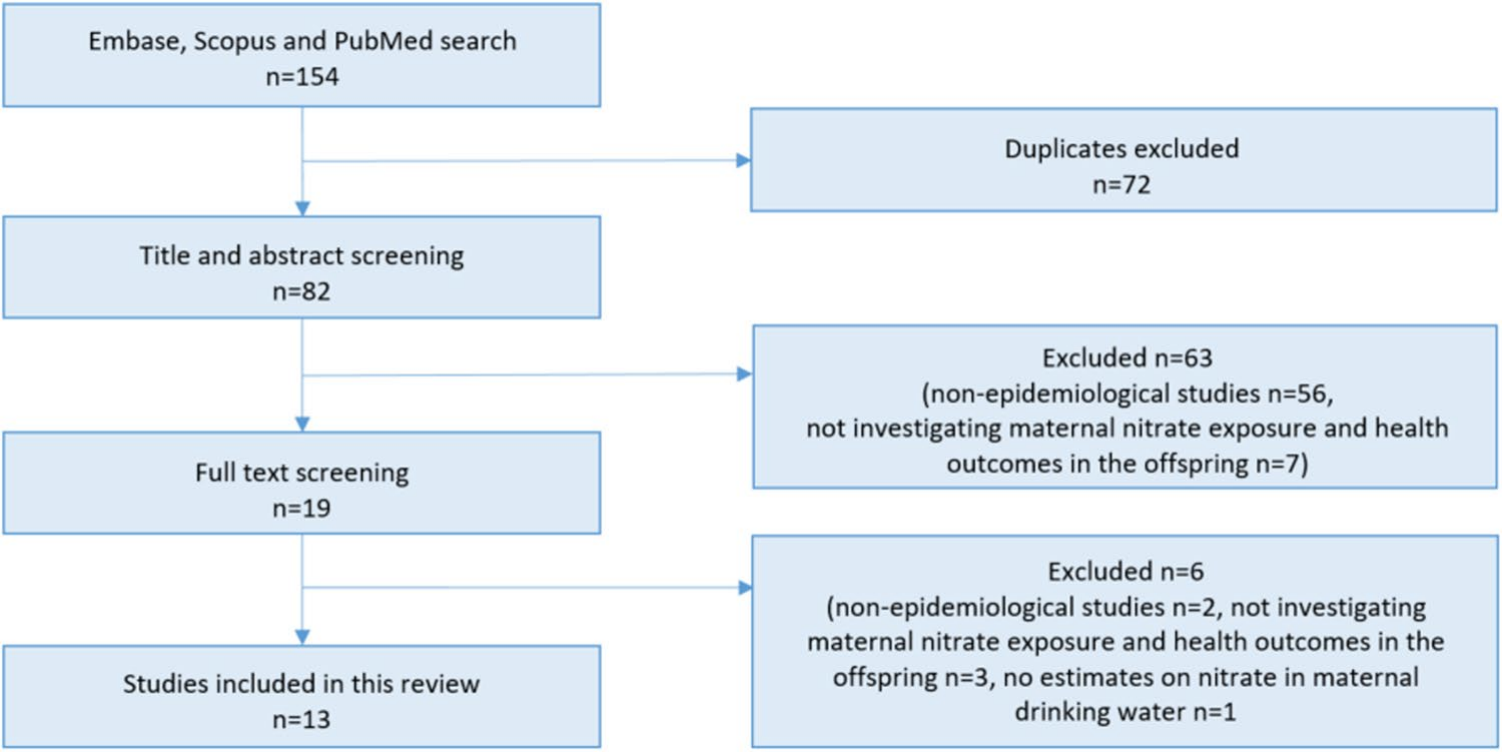
Illustration of the selection process of the included studies

We included epidemiological studies, published in English, in the period from January 2017 until December 12, 2022. This period was chosen because we wanted to include most recent publications and summarize the knowledge obtained since the review by Ward et al. from 2018. Only studies with estimates of nitrate in maternal drinking water were included. The selection process of the included papers is illustrated in Fig. 1. For each outcome, a summary of studies previously included in a literature review is provided at the end of each section.

## Results

We identified 13 studies that fulfilled our inclusion and exclusion criteria. The included studies are presented in Table 1 with a short description of study design, exposure characteristics, investigated outcomes, adjustment parameters, and main findings. The majority of studies focused on adverse birth outcomes (*n* = 9) [18–26], whereas health effects showing later in life were investigated in few studies (*n* = 4) [27–30]. Eight studies were conducted in Denmark, four studies were conducted in the USA, and one study was conducted in Italy, Spain, and Korea. The study participants were born between 1985 and 2017. Ten of the studies were cohort studies, two studies were case-control studies, and one study had an ecological design. The maternal exposure level was obtained by different approaches as described below.

**Table 1:** Characteristics of the included studies on prenatal exposure to nitrate and health effects

Author, year	Location	N	Study design	Exposure		Outcome	Adjustment for confounding variables in the main model	Main findings
				Origin of exposure information	Exposure level			
Pregnancy loss and stillbirth								
Ebdrup et al., 2022 [18]	Denmark	86,490	Cohort study	Household-level information on nitrate exposure from public water systems in the period of pregnancy	Median exposure 1.81 mg/L NO_3_^-^ and 95% prediction interval 0.17–18.74 mg/L NO_3_^-^	Pregnancy loss	The mother’s age, educational level, occupational status, body mass index, smoking, and alcohol intake together with population density	No consistent findings. When modeling the exposure as a continuous variable an association was found with pregnancy loss in first trimester for exposure levels from 1 to 10 mg/L with lower risk at higher exposure levels
Thomsen et al., 2021 [19]	Denmark	652,810	Cohort study	Household-level information on nitrate exposure from public water systems in the period of pregnancy	Exposed ≤ 1 mg/L NO_3_^−^: 23%. Exposed > 25 mg/L NO_3_^−^: 3.4%	Stillbirth	The mother’s body mass index, age, smoking, educational level, occupational status, parity, and urbanicity of the mother’s residential address	No observed association between nitrate in drinking water and risk of stillbirth when nitrosatable drug intake was not accounted for. The group exposed to secondary amines and nitrate exposure levels > 25 mg/L had a HR of 3.11 (95% CI, 1.08–8.94) for the risk of stillbirth
Preterm delivery and birth weight								
Huang et al., 2018 [20]	California (USA)	1,448,600	Cohort study	Nitrate exposure on a census tract level	A range from 0 to 85.48 ppm nitrate	Preterm delivery	Maternal race, age, and years of education and payer of delivery expenses	Higher risk of preterm delivery with an OR of 1.02 (95% CI, 1.01, 1.03) per 9.33 parts per million increase (IQR change) in nitrate exposure
Sherris et al., 2021 [21]	California (USA)	1,443,318 (discordant sibling sets *n* = 188,564)	Cohort study	Nitrate exposure from public water systems during pregnancy on community water system level	Study participants exposed above the drinking water standard of 10 mg N/L nitrate-nitrogen = 0.6%	Preterm delivery	The within-mother analyses were adjusted for interval between pregnancies, parity, and age of the mother	Associations between nitrate exposure and preterm delivery at gestational week 20–31 with an OR of 2.52 (95% CI, 1.49, 4.26) for mothers exposed above 10 mg N/L compared with below 5 mg N/L. A weaker association observed with preterm delivery at gestational week 32–36
Coffman et al., 2022 [22]	Denmark	1,009,189	Cohort study	Household-level information on nitrate exposure from public water systems in the period of pregnancy	Median exposure level 1.9 mg/L NO_3_^−^ [IQR 1.0–3.4]	Preterm delivery	The child’s sex and birth year, the pregnancy number, urbanicity, maternal age, smoking status, income, educational level, and employment status.	Higher risk of preterm delivery at higher nitrate exposure levels. When categorizing the outcome into extremely, very, or moderate preterm, the association was observed for moderate preterm with an OR in the continuous model of 1.02 (95% CI, 1.00, 1.03) per 10 mg/L increase in nitrate
Stayner et al., 2017 [23]	Ohio, Indiana, Iowa and Missouri (USA)	134,258	Ecologic study	County specific exposure levels with information on nitrate concentration in drinking water from public water systems	Mean nitrate exposure 0.95 ppm nitrate-nitrogen	Preterm delivery and low birthweight	Sex and race of the child, maternal educational level, income and smoking, state, year and season of birth, and population density	Nitrate exposure associated with very preterm delivery (birth before week 32) (RR of 1.08 (95% CI, 1.02, 1.15) per 1 mg N/L increase in nitrate-nitrogen exposure), and very low birth weight (< 1.5 kg) (RR of 1.17 (95% CI, 1.08, 1.25)) when restricting the analyses to counties with the percentage of private well users < 20%
Coffman et al., 2021 [24]	Denmark	852,348	Cohort study	Household level information on nitrate exposure from private and public water systems in the period of pregnancy	Median exposure 2.2 mg/L NO_3_^−^ and IQR 1.1–4.3 mg/L NO_3_^-^	Birth weight, body length and head circumference	The mother’s age, number of pregnancies, smoking, educational level, income, employment status. Region and urbanicity of the mothers address. Season, year of birth, and the child’s sex.	No association with term low birth weight. An inverse association between nitrate exposure and birth weight (9.7 g lower (95% CI, − 14.6, − 4.8) for 25 mg/L nitrate)
Birth defects								
Stayner et al., 2022 [25]	Denmark	1,018,914	Cohort study	Household-level information on nitrate exposure from public water systems in the period of pregnancy	In 0.2% of the cohort, the exposure level exceeded 50 mg/L NO_3_^-^	Birth defects	Parental socioeconomic factors (employment, income and education), age (mother and father), maternal smoking, the child’s sex, birth order, year, and weight	An association with eye birth defects with an OR in the highest exposed group (≥ 25 mg/L nitrate) of 1.29 (95% CI, 1.00, 1.66). Among mothers < 25 years of age an association was observed with nervous system and ear, face, and neck birth defects with higher nitrate levels
Blaisdell et al., 2019 [26]	Missouri (USA)	348,250	Cohort study	County-level information on nitrate exposure from public water systems in the period 12 months prior to birth	The median nitrate concentration ranged from 0.12-0.33 mg-N/L	Congenital malformations	Race, ethnicity, socioeconomic status (participation in a federal assistance program), and age of the mother	Higher risk of limb deficiencies with higher levels of nitrate exposure with a RR of 1.26 (95% CI, 1.05, 1.51) per 1 mg N/L increase in nitrate level
Cancer								
Stayner et al., 2021 [27]	Denmark	Leukemia: 596 cases, 52,308 controls. Lymphoma: 180 cases, 15,329 controls. CNS: 310 cases, 26,897 controls	Case-control study	Household-level information on nitrate exposure from public water systems in the period of pregnancy	Average exposure in 1991 5.2 mg/L NO_3_^-^ (SD = 8.1 mg/L) and in 2015 3.6 mg/L NO_3_^-^ (SD = 7.2 mg/L)	Childhood cancer	The child’s sex, date of birth, birth order and weight. The mother’s age, smoking, educational level, employment status and income. The father’s age. Urbanicity	No association with leukemia and lymphoma. For childhood central nervous system cancers, an OR of 1.65 (95% CI, 0.97, 2.81) was observed in the group exposed to > 25 mg/L
Zumel-Marne et al., 2021 [28]	Italy, Spain and Korea	85 cases and 343 controls	Case-control study	Exposure levels in tap water in the municipality of living in the year of the child’s birth	Median exposure 3.9 mg/L NO_3_^-^	Neuroepithelial brain tumors (diagnosed between the ages of 10-24 years)	Educational level of the parents. The models were further stratified on the child’s sex, age, and country	Higher odds of neuroepithelial tumors in study participants exposed to nitrate levels belonging to the second (OR 1.62 95% CI, 0.74, 3.53) and third tertile (OR 1.76 95% CI, 0.91, 3.41) compared with the first tertile
Reproductive health								
Clemmensen et al., 2022 [29]	Denmark	985	Cohort study	Household-level information on nitrate exposure from public water systems in the period of pregnancy	Median exposure 2.0 mg/L NO_3_^-^	Semen parameters, testes volume and reproductive hormones in adult sons	The mother’s age, smoking and educational level together with variables strongly associated with each specific outcome, e.g., spillage and abstinence time	No observed associations between maternal nitrate exposure and the measured outcomes
Clemmensen et al., 2022 [30]	Denmark	15,819	Cohort study	Household-level information on nitrate exposure from public water systems in the period of pregnancy	Median exposure 2.0 mg/L NO_3_^-^	Timing of puberty in sons and daughters	Cohabitation and educational level of parents, the mothers age, smoking and alcohol intake, body mass index, and age at menarche	No strong associations. The sons exposed to nitrate levels > 25 mg/L from maternal drinking water tended to obtain puberty earlier than sons exposed ≤ 1 mg/L with an average age difference of -1.2 months (95% CI, − 3.0, 0.6)

## Exposure Assessment

Although all studies relied on utilizing drinking water data from pre-existing monitoring programs of water quality, there are differences in their spatial and temporal resolution. Four studies studied nitrate measures in drinking water aggregated at the county, municipality, or census tract level [20, 23, 26, 28], while nine studies were able to pinpoint the nitrate level supplied to the geocoded maternal residential addresses [18, 19, 21, 22, 24, 25, 27, 29, 30]. Eight studies could distinguish whether an address was served by a public or private well [18, 19, 22, 24, 25, 27, 29, 30]. Whether the studies accounted for temporal changes in exposure, either due to nitrate concentrations changes at the residence or moving to a different residence during the exposure time window, is described in the below section.

The nitrate concentration was either reported as the nitrate ion (here denoted as mg/L), as nitrate-nitrogen (here denoted as mg N/L, to convert to nitrate ion concentrations multiply by a factor of 4.43), or as ppm (equivalent to mg/L) [15]. In this review, we report nitrate concentrations in the units used in the original studies.

### Water Supply Area Level

In the eight Danish studies that investigated different outcomes, a yearly nitrate concentration for each public waterworks’ distribution area was calculated based on longitudinal monitoring data from the waterworks [18, 19, 22, 24, 25, 27, 29–31]. From the Danish Civil Registration System, information on the mothers’ addresses was obtained. This information was used to obtain the mothers’ exposure level during pregnancy in the distribution area serving her address or addresses if she moved. The Danish studies were able to distinguish between public and private wells, and the fraction of private well users was around four percent [32•]. Nitrate measurements from private wells were sparse and inadequately monitored, and most studies excluded private well users from the main analyses.

Similarly, to the Danish studies, the study by Sherris et al. from California also used longitudinal monitoring data, measured quarterly or annually, to match maternal residence to the community water zones’ physical boundaries and calculated individual exposures; however, they did not take maternal residential movements during pregnancy into account and relied on address at birth [21].

### Administrative Area Levels

In another Californian study by Huang et al., the monitoring data from community water systems were aggregated on a census tract level [20]. Addresses outside community water systems were assumed to be supplied by unregulated small systems or private wells, for which groundwater nitrate concentrations within a 6 × 6 mile grid were used as an exposure approximation. Temporal changes in exposure were not considered [20]. In the study by Zumel-Marne et al., information on nitrate levels was aggregated on a municipality level with an annual resolution [28].

Two studies conducted in the Midwestern USA used monitoring data from public water systems to calculate the nitrate exposure level on a county level [23, 26]. A monthly average for each county was calculated using nitrate levels measured at the different water systems and with weighing of the nitrate levels from each water system by the population size served by the specific water system. No information about if the mother received her drinking water from a private well or a public supply was available. Restrictions to counties with < 20% and < 10% private wells were made in sub-analyses [23, 26]. The four studies that explored the exposure on administrative area levels assumed that the mother’s address at the time of birth represented the address in the entire pregnancy period.

## Adverse Birth Outcomes

We identified nine studies focusing on adverse birth outcomes (one study investigated two birth outcomes): One study investigated the risk of pregnancy loss [18], one study investigated the risk of stillbirth [19], four studies investigated the risk of preterm delivery [20–23], two studies investigated the risk of low birthweight [23, 24], and two studies investigated the risk of birth defects [25, 26].

### Pregnancy Loss and Stillbirth

The two included studies investigating pregnancy loss and stillbirth were cohort studies with household-level information on nitrate in drinking water. A pregnancy loss before gestational week 22 was investigated by Ebdrup et al. who used information on pregnancy loss provided by women enrolled in the Danish National Birth Cohort around gestational week 11 [18]. An early pregnancy loss that occurred before enrollment was therefore not included. No association was found when the exposure was modeled as a categorical variable. However, when the exposure was analyzed continuously, an association with pregnancy loss in first trimester was observed only for exposure range 1 to 10 mg/L but not higher. The study reported that maternal intake of nitrosatable drugs did not modify the association between nitrate in drinking water and pregnancy loss.

Thomsen et al*.* reported that nitrate in maternal drinking water was not associated with stillbirth, which was defined as birth of a non-vital fetus from gestational week 22 or later [19]. However, when further considering nitrosatable drug intake, the study reported an increased risk of stillbirth in the group with an intake of secondary amines and a nitrate exposure level > 25 mg/L (HR of 3.11 (95% CI, 1.08–8.94)).

### Papers on Pregnancy Loss and Stillbirth Previously Included in a Literature Review

None of the abovementioned studies investigated spontaneous abortions occurring before gestational week 11. Studies on spontaneous abortion and stillbirth conducted prior to the cutoff date for this review were sparse. A cluster analysis of four women living at addresses with nitrate levels above the regulatory limit and experiencing spontaneous abortions [33] together with a case-control study without an indication of a harmful effect of nitrate [34] were included in a review from 2005 by Ward et al. [35].

### Preterm Delivery

An association between nitrate exposure and preterm delivery was observed in all four studies included in this review [20–23]. The association was investigated in study populations residing in different geographical areas and investigated using different study designs and exposure assessment methods. Sherris et al. compared siblings with different prenatal exposure levels to nitrate estimated on a community water system level in a within-mother analysis [21]. The strongest associations were found in the within-mother analysis for preterm delivery at gestational week 20–31 with an OR of 1.47 (95% CI, 1.29, 1.67) for mothers exposed to nitrate levels of 5–10 mg N/L and an OR of 2.52 (95% CI, 1.49, 4.26) for mothers exposed above 10 mg N/L compared with below 5 mg N/L. The study reported a weaker association with preterm delivery at gestational week 32–36 [21]. The reported findings were consistent in several secondary analyses that investigated the association in mothers who did not change residence from one pregnancy to the other and with inclusion of non-sibling births. An ecological study by Stayner et al. did also report an association with very preterm delivery (birth before gestational week 32) with a RR of 1.08 (95% CI, 1.02, 1.15) per 1 mg N/L increase in nitrate-nitrogen exposure assessed on a county level [23], whereas no association (RR 1.01 (95% CI, 0.97, 1.06)) was observed for preterm delivery (birth before gestational week 37). In contrast to the findings from Sherris et al. and Stayner et al., the study by Coffman et al. reported the strongest association with preterm delivery to be from gestational week 32–36 with an OR in the continuous model of 1.02 (95% CI, 1.00, 1.03) per 10 mg/L increase in nitrate (measured as nitrate on a household level) [23]. For preterm delivery occurring from gestational week 28–31, the OR was 1.01 (95% CI, 0.97, 1.05), and for preterm delivery occurring from gestational week 20–27, the OR was 1.01 (95% CI, 0.94, 1.07). Huang et al. did not differentiate between preterm and very preterm delivery and reported an OR for preterm delivery compared with term birth of 1.02 (95% CI, 1.01, 1.03) per interquartile range increase in nitrate exposure (9.33 ppm on a census tract level) [20].

### Papers on Preterm Delivery Previously Included in a Literature Review

In previous reviews [3, 12, 35], results from earlier studies on preterm delivery were only available from three studies with mixed results. One of these studies showed an exposure-response relationship between nitrate and preterm births [36], while the others did not show an association [37, 38]. Further, one of these studies investigated exposure to a mixture of atrazine and nitrate and did not find an association with preterm birth [37].

### Birth Weight

No association between nitrate exposure and low birthweight (< 2.5 kg) among babies born at term (birth ≥ gestational week 37) was observed in studies performed by Stayner et al. and Coffman et al. [23, 24]; however, in the study by Stayner et al., an association with very low birth weight (< 1.5 kg) was observed without restriction to term birth and with restriction to counties with < 20% private well users: The study reported a RR of 1.17 (95% CI, 1.08, 1.25) for very low birth weight per 1 mg N/L nitrate-nitrogen exposure (assessed on a county level). In the study by Coffman et al., the study reported a significant trend of decreasing birth weight with increasing nitrate exposure levels [24]. In the continuous model, birth weight was 9.7 g lower (95% CI, − 14.6, − 4.8) for 25 mg/L nitrate. Also, an association with nitrate exposure and shorter body length of the child was observed whereas head circumference was not associated with nitrate exposure.

### Papers on Birth Weight Previously Included in a Literature Review

Only one study was included in the prior reviews which indicated an association between nitrate exposure and lower birth weight at term [36]. Additionally, one study found an association between nitrate exposure and small for gestational age [39].

### Birth Defects

In a population-based study, Stayner et al. investigated the risk of birth defects in more than one million births in Denmark [25]. Information on birth defects diagnosed from birth until 2 years of age in the offspring was obtained from Danish registers, and the maternal nitrate exposure was evaluated on a household level. The study reported an association with eye birth defects with an OR in the highest exposed group (≥ 25 mg/L nitrate) of 1.29 (95% CI, 1.00, 1.66), while an inverse exposure-response relationship was observed when investigating any birth defects as one outcome possible due to live birth bias (described in “Discussion and conclusions” section). Among mothers younger than 25 years of age, they found a strong association with nervous system and ear, face, and neck defects for every 10 mg/L increase in nitrate exposure in maternal tap water. The authors suggested that this finding might be due to lifestyle behaviors such as increased use of folate among older mothers or co-occurring environmental exposures in young women. A restriction to mothers exposed below the drinking water standard did not change the results. Blaisdell et al. investigated the risk of birth defects reported in the first year of life in relation to nitrate exposure assessed on a county level in the period 12 months prior to birth [26]. The study reported a positive association with limb deficiencies with a RR of 1.26 (95% CI, 1.05, 1.51) per 1 mg N/L increase in nitrate level (measured as nitrate-nitrogen) and a weak association with heart and neural tube defects; an association between nitrate exposure and limb deficiencies or heart defects was not observed in the study performed by Stayner et al. [25].

### Papers on Birth Defects Previously Included in a Literature Review

Ward et al. summarized previously performed studies on birth defects [3••]. Five of six studies had found an association between nitrate in drinking water and the risk of neural tube defects or central nervous system defects combined. Positive associations were also observed for limb deficiencies and oral cleft defects within a study in the US National Birth Defects Prevention study [3••].

## Delayed Effects on Health Outcomes

We identified four studies investigating the association between prenatal exposure to nitrate and health effects later in life [27–30]. Two studies investigated the risk of childhood cancer, and two studies investigated the potential association with reproductive health including semen quality in adult men and timing of puberty.

### Childhood Cancer

Stayner et al. used a population based case-control design with household-level exposure information to investigate the risk of childhood cancer in children < 15 years of age [27]. Several exposure time windows from preconception to childhood were investigated. For the prenatal exposure window, no association with leukemia or lymphoma was observed whereas an association with central nervous system cancers was found in the highest exposed group exposed > 25 mg/L nitrate with an OR of 1.65 (95% CI, 0.97, 2.81). This association was also observed for the preconception exposure period with an OR of 1.82 (95% CI, 1.09, 3.04). In the study by Zumel-Marne et al., the authors investigated if prenatal exposure on a municipality level was associated with neuroepithelial brain tumors (excluding midbrain tumors) in children aged 10–24 years [28]. They found higher odds of neuroepithelial brain tumors in the second exposure tertile (3.27–8.47 mg/L) (OR 1.62 95% CI, 0.74, 3.53) and third exposure tertile (≥ 8.48 mg/L) (OR 1.76 95% CI, 0.91, 3.41) compared with the first tertile (≤ 3.26 mg/L) with a weak indication of a dose-dependent increase in odds (*p* for trend 0.12).

### Papers on Childhood Cancer Previously Included in a Literature Review

Studies on childhood brain cancer that were published before 2017 and therefore not included in the systematic literature search for this review used different ways of measuring nitrate exposure during pregnancy and the results from these studies are mixed [40–42]. One study found an association in one out of three regions with consumption of water from private wells which were known to have higher levels of nitrate than water from public wells [42]. Another multi-country study found an association between private well use and childhood brain cancer in two of the seven regions included in the study [40]. Finally, one study investigated the risk of death from childhood brain cancer and found a higher odds of death in the high nitrate exposed group (> 0.31 mg N/L) compared with the low exposed group (≤ 0.31 mg N/L) [41].

### Reproductive Health

Clemmensen et al. investigated if prenatal exposure to nitrate was associated with adult male fecundity among 985 young men with low exposures (median nitrate exposure 2.0 mg/L) who delivered a semen sample and a blood sample [29]. No associations with measures of male fecundity were observed at these exposure levels. Another study by Clemmensen et al. investigated if nitrate in maternal drinking water was associated with timing of puberty [30]. The participants gave information on their pubertal developmental stage from the time they turned 11 years of age until the end of puberty. Besides household-level information on nitrate in drinking water, the study included information on maternal nitrate intake from diet and antioxidant intake during pregnancy. Nitrate exposure levels from diet and drinking water were not associated. The study did not find a clear association between nitrate exposure and timing of puberty; however, males exposed to > 25 mg/L tended to achieve pubertal milestones earlier than males exposed to ≤ 1 mg/L (a combined pubertal marker was obtained 1.2 months earlier (95% CI, − 3.0, 0.6) for males exposed to > 25 mg/L compared to males exposed to ≤ 1 mg/L). No other drinking water contaminants were considered; however, adjustment for population density was used to account for environmental exposures related to place of living.

## Discussion and Conclusions

The included studies provided some support to the hypothesis that nitrate in maternal drinking water may have some adverse health effects in the offspring, including preterm delivery, lower birth weight, and birth defects, also at drinking water levels below the current European and US drinking water standards. For stillbirth, an association was observed only when intake of nitrosatable drugs were considered. Four studies investigated health outcomes appearing later in life and found no associations with childhood lymphoma and leukemia, whereas associations with central nervous system cancers were observed. For reproductive health outcomes, no association with male fecundity and only a suggestive association with timing of puberty in males and not females were observed. There was a relatively small number of studies available for most of the outcomes, and somewhat conflicting results for some outcomes across the studies. Further, several studies investigated multiple outcomes that may increase the risk of chance findings.

When investigating health effects in late pregnancy or after birth, studies conditioned on survival until this period. For this review, we did not identify recent studies investigating the risk of miscarriage occurring before gestational week 11. An effect of nitrate resulting in early miscarriage may bias the studied associations towards no association between nitrate and the other outcomes investigated [43•]. This may also explain the inverse exposure-response relationship observed for several of the studied birth defects in the study by Stayner et al. [25], and the lower risk of miscarriage at higher exposure levels in the study by Ebdrup et al. [18].

The majority of studies were conducted in the USA or in Denmark where public water systems have to meet existing drinking water standards [8, 9]. In the USA in 2016, it was estimated that 0.32% of the drinking water systems exceeded the drinking water standards [8], and in Denmark in 2012, public supply wells exceeded the drinking water standards in three out of 10,000 consumers [44]. Study participants were generally exposed far below the drinking water standards, which has to be taken into account when considering the importance of the findings. Nitrate exposure levels across the world have been reviewed by IARC, and some of the included studies have found high levels of nitrate contamination. A study on 53 African wells found that 23% of the samples had nitrate-nitrogen levels above 11.3 mg N/L [1]. Private well users are also generally exposed to higher nitrate levels than observed in these studies. This is true for private well users in the USA and Denmark, too [3, 32, 32, 2, 3

Exposure assessment in four out of the 13 studies was based on geographic areas that were not necessarily identical with the physical boundaries of the water distribution systems. Instead, the nitrate measurements from public water systems in these administrative areas were aggregated. If the mother lived in one of these areas, she was assigned the aggregated estimated exposure level of the entire area, without taking into account if she was actually served by the water system, where the measurements were obtained. This results in a risk for misclassification if nitrate levels within the used geographic unit vary. All studies except those in Denmark estimated exposure at the place of birth, and assumed that women did not move during their pregnancies which was not the case in the study by Ebdrup et al. where movements were reported in nine percent of pregnancies [18]. Furthermore, most of the studies did not include information on maternal intake of tap water/bottled water. These issues would result in misclassification of the exposure, which if non-differential with respect to the outcomes would be expected to give rise to bias towards the null. All studies based their exposure-level assessment on nitrate measurements collected during pregnancy, and the risk of differential misclassification of the exposure is considered low. Temporal changes in nitrate concentration in groundwater-based water supply systems are reported to occur slowly [45]; however, if the nitrate concentration is measured with broad time intervals or the water supply system is based on surface water where seasonal variation exists, this could add to misclassification of the exposure [2].

Associations between nitrate concentration in drinking water and socioeconomic status have been described [46]. Socioeconomic status might therefore be a confounding variable if it is also associated with the studied outcome [47]: All the studies included in this review adjusted the main analyses for proxies for socioeconomic factors; however, residual confounding might occur. Several of the studies adjusted the main analyses for urbanicity or population density to account for socioeconomic differences and other environmental exposures, like air pollution, related to place of living. None of the studies adjusted for co-occurring contaminants in drinking water that could be associated with nitrate exposure and be a confounding variable if it is also associated with the outcome.

Two studies included information on nitrosatable drug intake, and one of them found nitrate to modify the association between nitrosatable drug intake and stillbirth [18, 19]. Only one study included information on antioxidant intake and nitrate intake from diet to study if nitrate intake from the two different sources (drinking water and diet) had a different impact on the outcome and whether antioxidants modified a potential association [30]. Previously performed studies have associated nitrosatable drug intake and dietary nitrate and nitrite intake with adverse birth outcomes [48–51]. The study by Clemmensen et al. did not find nitrate intake from diet and drinking water to be associated with each other [30]; however, in other populations, nitrate intake from diet might be a confounding variable that should be considered for inclusion in the studies. Further, investigating nitrate intake from diet, antioxidant intake, and nitrosatable drug intake together with nitrate intake from drinking water might help to reveal the relevant mechanistic pathways of nitrate exposure, where the formation of NOCs is affected by these components. Including this information in future studies will further strengthen the ability to draw causal inferences on a potential association between prenatal exposure to nitrate and health effects.

In this review, the most consistent results were observed for preterm delivery where all four included studies observed an association with prenatal nitrate exposure, although it differed in which gestational week the risk of preterm delivery was present. Given the small number of studies for each of the studied outcomes and some inconsistencies in the study results, a firm conclusion on the health impact of prenatal exposure to nitrate cannot yet be drawn. However, findings from our review in which associations with preterm delivery, birth defects, birth weight, and childhood brain cancer were described are of serious concern and reveal a need for further research on this topic. In addition, studies on the risk of miscarriage in early pregnancy will further clarify if there is a risk of live-born bias in studies on prenatal exposure to nitrate.

## Abbreviations

*IARC*: International Agency for Research on Cancer
*NOCs*: *N*-nitroso-compounds
*MCL*: maximum contaminant level
